# 造血干细胞移植后静脉血栓栓塞症诊断与防治中国专家共识（2022年版）

**DOI:** 10.3760/cma.j.issn.0253-2727.2022.03.002

**Published:** 2022-03

**Authors:** 中华医学会血液学分会 中国医师协会血液科医师分会

造血干细胞移植（HSCT）是多种良性和恶性血液病的重要根治手段，得到越来越广泛的应用。静脉血栓栓塞症（venous thromboembolism, VTE）是HSCT后常见的严重并发症，不仅降低了HSCT患者的生存质量，同时也降低了HSCT患者的长期生存率[1]–[2]。HSCT后VTE主要包括深静脉血栓形成（deep venous thrombosis, DVT）、肺血栓栓塞症（pulmonary thromboembolism, PTE）、导管相关血栓形成（catheter-related venous thrombosis, CRT）和浅静脉血栓形成（superficial vein thrombosis, SVT）等。HSCT患者VTE发生率升高与恶性疾病本身、免疫抑制及免疫紊乱状态、高感染风险、合并用药多等诸多因素有关。中华医学会血液学分会及中国医师协会血液科医师分会组织国内血液科相关专家制定本共识，旨在进一步规范HSCT后VTE的诊断、预防与治疗。本共识不涉及移植后血栓性微血管病及肝窦隙阻塞综合征相关内容。

一、HSCT后VTE的流行病学特征

受不同定义、不同人群等因素影响，HSCT后VTE的发生率为0.5％～23.5％[1]–[9]。荟萃分析显示，HSCT后VTE的总体发生率为5％，自体造血干细胞移植（auto-HSCT）、异基因造血干细胞移植（allo-HSCT）后VTE的发生率分别为4％（1％～15％）、4％（2％～6％）[10]。auto-HSCT、allo-HSCT患者VTE发生风险分别是一般人群的2.6倍[8]、7.3倍[11]。HSCT后VTE大多发生于造血干细胞植入后，中位发生时间为移植后98.5～480 d[1],[3]–[4],[12]。由于深静脉置管的普及，HSCT后最常见的VTE类型为CRT，其次为DVT，PTE相对少见。allo-HSCT后VTE中约50％为CRT，DVT占40％左右，另外不到10％为PTE或PTE合并DVT[1]。

CRT涉及的深静脉置管主要是导管末端位于腔静脉（上腔静脉和下腔静脉）的中心静脉通路装置（central venous access device, CVAD），包括中心静脉导管（central venous catheter, CVC）、经外周置入中心静脉导管（peripherally inserted central catheter, PICC）和输液港。血栓可在输液导管内部、周围或尖端形成。在本共识中，CRT特指导致置管侧静脉管腔的部分或完全阻塞性DVT或SVT。HSCT后有症状CRT的发生率为1.38％～8.00％[2],[8],[13]。

二、HSCT后VTE的发病机制与危险因素

（一）发病机制

能够直接或间接影响血管内皮细胞损伤、血流淤滞、血液高凝状态的各种病理生理变化都可以导致VTE发生。HSCT预处理过程中放疗或化疗，使用粒细胞集落刺激因子（G-CSF）、糖皮质激素或钙调磷酸酶抑制剂，以及移植物抗宿主病（GVHD）等都可能引起内皮细胞损伤[14]–[15]。HSCT过程中，尤其allo-HSCT，会出现促凝因子升高、抗凝蛋白下降，以及血小板过度激活，形成“获得性”高凝状态[16]–[17]。如果恶性血液病持续存在或复发，肿瘤细胞会释放组织因子和促凝蛋白，进一步加重血液“高凝状态”[18]。HSCT患者住院时间延长、卧床和活动减少，以及留置深静脉置管等因素都可能引起血液瘀滞[19]–[20]。这些因素的共同作用可导致HSCT后VTE的发生。

（二）危险因素

allo-HSCT[3],[19]、清髓性预处理和免疫调节药物[21]、GVHD[3],[6]–[7],[19]、感染[19]、既往VTE病史[1],[4],[6],[14]、留置静脉输液导管[20]、恶性血液病未缓解或复发[1]、住院卧床时间长[19]以及使用大剂量糖皮质激素[2]均可导致HSCT后VTE发生风险升高。

三、HSCT后VTE的诊断

（一）非导管相关DVT的诊断[22]–[23]

1. 临床评估：HSCT后出现颜面、颈部或锁骨上区局限水肿，单侧肢体肿胀、疼痛或沉重感，无法解释的持续小腿痉挛、疼痛等症状/体征应疑诊DVT，按图1所示完成诊断流程。需询问的病史及用药史包括目前原发病状态、移植类型及移植后所处阶段、移植并发症、当前用药、既往VTE病史、VTE家族史、有无正在或近期曾使用促凝药物（如口服避孕药）等；体格检查包括出现症状区的局部情况、血压、心率、血氧饱和度、体温；化验检查包括血常规、凝血酶原时间（PT）、活化部分凝血活酶时间（APTT）、纤维蛋白原、D-二聚体、纤维蛋白降解产物（FDP）和肝肾功能等。临床可疑DVT的患者，需进一步完善影像学检查协助诊断，但如影像学检查难以立即完成，可根据患者的基本情况，进行VTE可能性的Wells评分（表1）[24]，并结合D-二聚体结果确定进一步的检查方案。如Wells评分<2分，且D-二聚体阴性，可排除DVT；如Wells评分≥2分和（或）D-二聚体阳性，均需进一步影像学检查。

**图1:**
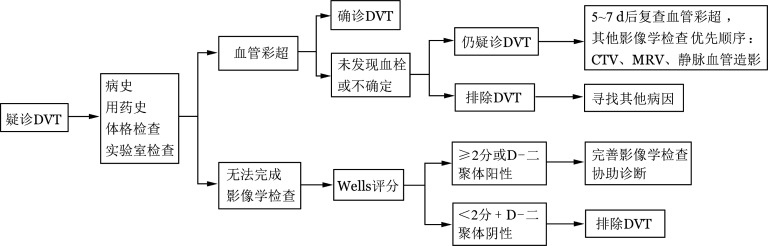
非导管相关深静脉血栓形成（DVT）筛查流程 CTV：CT静脉成像；MRV：磁共振静脉成像

**表1:** 评估深静脉血栓形成（DVT）临床可能性的Wells评分[24]

项目	评分
活动性恶性肿瘤（近6个月内接受肿瘤治疗或目前正采取姑息疗法）	1
下肢麻痹、瘫痪，或下肢石膏固定	1
4周内卧床≥3 d或4周内大手术史	1
沿深静脉系统走行的局部压痛	1
下肢肿胀	1
胫骨结节下方10 cm处腿围较对侧增加≥3 cm	1
患肢可凹性水肿	1
浅静脉侧支循环（非静脉曲张）	1
其他比DVT更符合的诊断	−2

注：如果双侧下肢均有症状，以症状严重侧为准

2. 影像学检查：确诊DVT检查包括血管彩色多普勒超声、CT静脉成像（CTV）、磁共振静脉成像（MRV）和静脉血管造影。血管彩色多普勒超声（以下简称血管彩超）因其无创、便捷和准确性高，是DVT诊断的首选影像学检查。如单次血管彩超阴性，但临床仍怀疑DVT，则5～7 d后重复血管彩超检查。如果临床高度可疑，但超声检查结果阴性或不确定，或持续高度怀疑近端DVT（如下腔静脉、髂静脉），可立即行CTV、MRV或静脉造影确诊。静脉造影是确诊DVT的“金标准”，但因其有创及造影剂可加重肾损害，不推荐作为常规检查。

3. 进一步检查：诊断DVT的患者，根据临床症状和体征，必要时进行肺血栓栓塞症（PTE）监测及筛查。既往有VTE病史或家族史的患者应进行遗传性易栓症相关基因及蛋白活性检测。

（二）PTE的诊断[25]–[26]

移植后出现不明原因胸闷气短、胸痛、呼吸急促、心动过速、烦躁、咯血、晕厥或低氧血症等表现，尤其同时存在DVT的症状或近期有DVT病史者应疑诊PTE，具体诊断流程见图2。

**图2:**
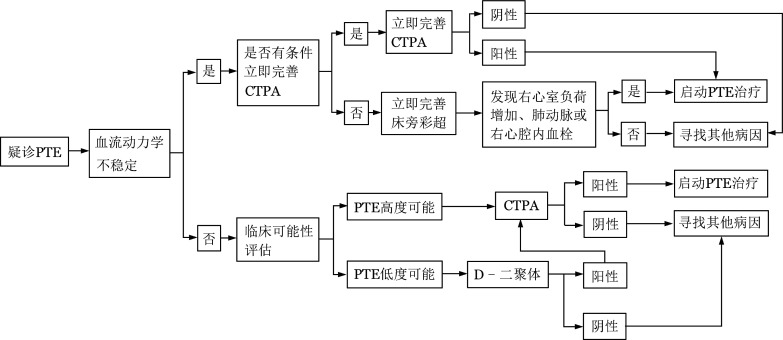
肺血栓栓塞症（PTE）诊断流程 CTPA：CT肺动脉造影

1. 临床评估：①病史询问及体格检查：除上述可疑DVT时的病史询问外，还需注意是否存在或近期有DVT病史。完成体温、呼吸频率、肺呼吸音、心音、血压、心率、血氧饱和度、检查四肢是否有肿胀等体格检查。②基本辅助检查：血常规、肝肾功能、凝血功能（PT、APTT、D-二聚体）、血气分析、B型钠尿肽（BNP）或N末端脑钠肽前体（NT-proBNP）、肌钙蛋白I/肌钙蛋白T（cTNI/cTNT）、心电图等。③临床可能性评估：包括Wells评分[27]和修正Geneva评分[28]（附表1）。本共识建议均采用简化版，临床容易操作。

**附表1:** 肺血栓栓塞症（PTE）临床可能性评估

评分系统	评分
简化修订版Geneva评分[28]	
肺栓塞或DVT病史	1
过去1个月内手术或骨折	1
活动性肿瘤	1
心率	
75~94次/min	1
≥95次/min	2
咯血	1
单侧下肢疼痛	1
下肢深静脉触痛及单侧下肢水肿	1
>65岁	1
简化Wells评分[27]	
肺栓塞或DVT病史	1
过去4周内有制动或手术史	1
活动性肿瘤	1
心率≥100次/分	1
咯血	1
DVT症状或体征	1
其他鉴别诊断可能性低于肺栓塞	1

注：DVT：深静脉血栓形成。临床可能性：简化修订版Geneva评分：0～2分为不可能，≥3分为可能；简化Wells评分：0～1分为低度可能，≥2分为高度可能

2. 影像学检查需要注意的问题：确诊PTE首选CT肺动脉造影（CTPA），但CTPA对亚段及亚段以下肺动脉血栓的敏感性较差。核素肺通气/灌注（V/Q）显像不受肺动脉直径影响，对诊断亚段以下肺动脉内血栓有特殊意义，但不能区分导致肺血流或通气受损的因素，如肿瘤、炎症、慢性阻塞性肺病等，适用于CTPA有禁忌的患者。如CTPA、V/Q显像都难以进行，可选择磁共振肺动脉造影（MRPA）。选择性肺动脉造影为诊断PTE的“金标准”，但因其有创及CTPA的发展和完善，不作为常规推荐。另外，所有PTE患者都应完善四肢血管彩超除外DVT。

3. 危险分层：具体见图3，根据是否合并血流动力学不稳定，分为高危和非高危PTE[26]。非高危PTE根据肺栓塞严重指数（PESI）[29]或简化版PESI（sPESI）[30]分为中危和低危PTE。本共识建议采用更易操作的sPESI（附表2）。中危PTE根据是否合并右心室功能不全和心脏生物学标志物升高，进一步划分为中高危和中低危PTE。

**图3:**
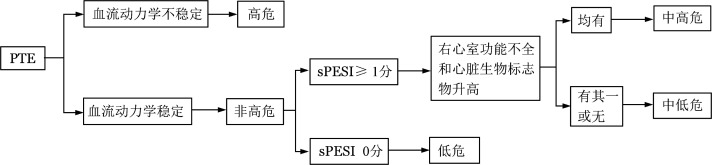
肺血栓栓塞症（PTE）危险分层 血流动力学不稳定：①需要心肺复苏；或②休克：收缩压<90 mmHg或需升压药维持血压，且有脏器灌注不足的表现；或③收缩压<90 mmHg/较基础血压下降幅度>40 mmHg，持续15 min以上，且排除感染中毒性休克、新发心律失常或低灌注等引起。右心室功能不全诊断标准：超声检查符合下述表现：①右心室扩张（右心室舒张末期内径/左心室舒张末期内径>1.0或0.9）；②右心室游离壁运动幅度减低；③三尖瓣反流速度增快；④三尖瓣环收缩期位移减低（<17 mm）；或CTPA检查四腔心层面发现右心室扩张（右心室舒张末期内径/左心室舒张末期内径>1.0或0.9）。心脏生物标志物包括：①心肌损伤标志物心脏肌钙蛋白T、心脏肌钙蛋白I；②心衰标志物B型钠尿肽（BNP）、N末端脑钠肽前体（NT-proBNP）。sPESI：简化版肺栓塞严重指数

**附表2:** 简化版肺栓塞严重指数（sPESI）[30]

项目	评分
年龄>80岁	1
恶性肿瘤	1
慢性心力衰竭或慢性肺部疾病	1
脉搏≥110次/分	1
收缩压<100 mmHg	1
动脉血氧饱和度<90%	1

注：危险分层：0分为低危，≥1分为中危

（三）HSCT后CRT的诊断[31]

1. 临床表现：多数HSCT患者的CRT无症状，仅1/3有明显症状和/或体征[32]。CRT根据血栓发生部分，分为导管相关DVT和SVT。导管相关DVT表现为置管侧肢体、颈部、肩部、胸部、颜面部水肿，伴或不伴受累部位疼痛、皮温升高、浅表静脉显露、颈部或肢体运动障碍、肢体红斑或麻木感等；导管相关SVT表现为沿浅静脉走行的压痛、红斑、局部发热及压痛等，临床表现类似于炎症过程，也被称为“血栓性静脉炎”。

2. 影像学检查：首选血管彩超，可用于检测导管到达的静脉血栓（如颈静脉、腋静脉和锁骨下静脉远端），CTV或MRV对于上/下腔静脉、髂总静脉、锁骨下静脉、无名静脉血栓的诊断有价值，还可发现并存的血管外压迫因素，如肿瘤、胸廓出口压迫等。血管造影是诊断CRT的“金标准”，但因其有创，不作常规推荐。

（四）HSCT后VTE鉴别诊断

1. 感染：HSCT患者感染风险高，在疑诊VTE的患者中应注意感染排查。建议同时完善C反应蛋白（CRP）、降钙素原（PCT）等检查，评估患者是否有发热、皮温升高等，以排除感染（尤其导管相关感染）。PTE需与肺部感染等进行鉴别，DVT需与蜂窝织炎、淋巴管炎等鉴别。

2. 其他原因引起的导管梗阻：推荐首先检查导管是否存在机械性阻塞，如管线扭转、打结、导管尖端紧贴血管壁、导管尖端错位、导管周围纤维鞘形成或导管内药物残留堵塞导管等，同时行血管彩超或胸部X线检查协助诊断。

3. 其他原因引起的肢体水肿或活动障碍：肌肉损伤、慢性水肿、慢性静脉功能不全、神经系统病变等。

四、HSCT后VTE的预防

不建议HSCT后常规预防性抗凝，尤其合并血小板减少时。血小板计数恢复后，是否给予预防性抗凝，应根据各中心经验、患者基础疾病及危险因素等确定。

目前尚无HSCT后预防性抗凝的大样本临床研究数据资料。接受化疗肿瘤患者的前瞻随机对照研究显示，预防性抗凝可降低VTE风险，但不能改善患者的总体生存[33]–[34]。血小板减少及出血在是HSCT后更为常见[35]–[36]，抗凝治疗是HSCT患者发生出血事件的独立危险因素[7]。研究显示，HSCT前有VTE病史的患者，在血小板植入前暂停抗凝不增加HSCT早期VTE复发风险[37]。各种抗凝药物在HSCT后的安全性及有效性有待积累更多证据加以验证。如需预防性抗凝，可根据各中心经验进行药物选择，可供参考的药物用法及用量见附表3。

**附表3:** 静脉血栓栓塞症（VTE）抗凝药物预防方案

药物名称	用法与用量
普通肝素	5000 U每8~12 h 1次，皮下注射
依诺肝素	40 mg每日1次，皮下注射
达肝素钠	5000 U每日1次，皮下注射
磺达肝癸钠	2.5 mg每日1次，皮下注射
利伐沙班	10 mg每日1次，口服

（一）allo-HSCT后VTE预防

allo-HSCT后VTE风险升高且与出血风险并存。VTE的危险因素（如GVHD、机会感染）既可导致VTE风险升高，也导致血小板减少和出血风险升高[3],[7]，在临床诊疗中需综合考虑患者的VTE及出血风险，个体化确定VTE监测频率及是否预防性抗凝。目前关于allo-HSCT后VTE风险的预测模型包括针对移植早期患者的HIGH-2-LOW[38]（表2）和针对移植晚期期患者的BMTSS-HiGHS2[11]（表3），但上述预测模型均需大样本临床研究进一步验证。

**表2:** allo-HSCT患者HIGH-2-LOW静脉血栓栓塞症（VTE）预测模型[38]

危险因素	积分
导管相关DVT病史	1
住院患者（移植后30 d）	1
Ⅲ/Ⅳ度GVHD（移植后30 d）	1
PE或下肢DVT病史	2
淋巴瘤患者	1
肥胖（BMI≥35 kg/m^2^）	1
WBC≥11×10^9^/L（+30 d）	1

注：GVHD：移植物抗宿主病；PE：肺栓塞；DVT：深静脉血栓；BMI：体质指数。移植后30 d进行评分，≥2分为高危，1分为中危，0分为低危

**表3:** allo-HSCT患者BMTSS-HiGHS2静脉血栓栓塞症（VTE）预测模型[11]

危险因素	评分
脑卒中病史	3
慢性GVHD	1
高血压	2
男性	2
干细胞来源为外周血干细胞	3

注：GVHD：移植物抗宿主病。移植后2年进行评分，≥5分为高危，<5分为低危

1. HIGH-2-LOW模型：此模型是针对移植早期患者的VTE风险预测模型。参照此模型，在移植后30 d对allo-HSCT患者的VTE发生风险进行评估，预测移植后100 d内VTE发生风险。评分≥2分为高危，1分为中危，0分为低危。未行没有预防性抗凝治疗时，HIGH-2-LOW评分高危患者移植后100 d VTE发生率高达10.25％，中危患者为3.58％，低危患者为1.54％[38]。HSCT前有VTE病史，HIGH-2-LOW评分中危或高危的患者，在血小板植入后给予抗凝治疗，可减少移植后100 d内VTE复发[37]。

2. BMTSS-HiGHS2模型：此模型是针对移植晚期患者的VTE风险预测模型，参照此模型，在移植后2年对allo-HSCT患者的VTE发生风险进行评分，预测移植晚期VTE风险。评分≥5分为高危，<5分为低危。高危患者移植后5年、10年的VTE累积发生率分别为（5.3±1.0）％、（9.3±1.5）％，低危患者分别为（0.9±0.3）％、（2.4±0.5）％[11]。

对于HIGH-2-LOW或BMTSS-HiGHS2模型评分VTE风险高危的allo-HSCT患者，需密切监测VTE的发生，根据患者既往VTE病史、基础疾病、血小板恢复情况、出血风险等确定是否给予预防性抗凝。

（二）多发性骨髓瘤（multiple myeloma, MM）患者auto-HSCT后VTE预防

建议MM患者接受auto-HSCT后，根据VTE风险评分进行VTE预防。

MM患者血栓风险升高，尤其接受沙利度胺、来那度胺等免疫调节剂（Immunomodulatory drugs, IMiD）维持治疗的患者，VTE发生率高达30％[9]。目前缺乏MM接受auto-HSCT后VTE风险预测模型。基于VTE预防在接受IMiD治疗的新诊断MM的获益，建议有条件的单位可参考新诊断MM患者VTE风险预测的SAVED评分（表4）[39]和IMPEDE评分（表5）[40]；高危患者（SAVED评分≥2分或IMPEDE评分>3分）应进行VTE预防［可用低分子量肝素（LMWH）或直接口服抗凝剂（DOAC）］；低危患者（SAVED评分<2分或IMPEDE VTE评分≤ 3分）可不预防或仅口服阿司匹林预防。

**表4:** SAVED静脉血栓栓塞症（VTE）风险评分[39]

变量	积分
90 d内外科手术	+2
亚洲人	-3
VTE病史	+3
年龄≥80岁	+1
地塞米松	
标准剂量（每周期120~160 mg）	+1
大剂量（每周期>160 mg）	+2

**表5:** IMPEDE静脉血栓栓塞症（VTE）风险评分[40]

变量	积分
免疫调节剂（IMiD）治疗	+4
肥胖（BMI≥25 kg/m^2^）	+1
骨盆、髋部或股骨骨折	+4
使用促红细胞生成素	+1
低剂量地塞米松（每月≤160 mg）	+2
大剂量地塞米松（每月>160 mg）	+4
多柔比星或多药化疗	+3
亚洲/太平洋岛民	-3
既往VTE病史	+5
中心静脉置管或输液港	+2
正在进行血栓预防：治疗量LMWH或华法林	-4
正在进行血栓预防：预防量LMWH或阿司匹林	-3

注：BMI：体质指数；LMWH：低分子量肝素

（三）HSCT后CRT的预防

不推荐HSCT后留置输液导管的患者常规进行预防性抗凝。

目前，前瞻随机对照研究未能证实预防性抗凝对CRT预防有益，且可能增加出血风险[41]。CRT发生的危险因素见表6[2],[42]。CRT的预防首先包括适当运动及置管侧肢体的活动及导管相关危险因素的控制[43]。有CRT发生危险因素时，应重视CRT的临床症状、体征监测。无CRT症状、体征的患者，不推荐进行常规血管彩超监测。

**表6:** 导管相关血栓形成（CRT）的危险因素[2],[42]

设备相关危险因素（高危>低危）
PICC>CVC>输液港
CVC置管静脉：股静脉>颈内静脉>锁骨下静脉
PICC置管静脉：
头静脉>贵要静脉或肱静脉
左侧置管>右侧置管
导管直径：6F>5F>4F；多腔>单腔
导管外径与置管静脉内径比值≤0.45
导管尖端位置：上腔静脉下1/3或右心房与上腔静脉交界区>上腔静脉上段
患者相关危险因素
恶性血液病活动期
近30 d内手术或外伤
既往VTE病史
终末期肾病
高龄
30 d内制动
导管相关血流感染
治疗相关危险因素
输注两性霉素B
肠外营养
输注化疗药
糖皮质激素累积量大

注：CVC：中心静脉导管；PICC：经外周置入中心静脉导管；VTE：静脉血栓栓塞症

五、HSCT后VTE的治疗

（一）HSCT后非导管相关DVT的治疗[22]–[23],[44]

无抗凝禁忌证的患者，一旦确诊急性DVT（2周内），应立即开始抗凝治疗。抗凝治疗无效的患者、有肢体丧失风险或严重难治性近端血栓形成者，如排除溶栓禁忌证，可予导管内直接溶栓。出血风险高或有溶栓禁忌证的患者，可予经皮机械取栓。选择导管内直接溶栓或经皮机械取栓，需血液科、介入科或血管外科专家共同决策。

有抗凝禁忌证的患者：①急性近端下肢（骨盆静脉/髂静脉/下腔静脉/股静脉/腘静脉）DVT：选择放置临时下腔静脉滤器（IVC），待抗凝禁忌证解除后予以抗凝治疗；②急性远端下肢DVT（腓静脉/胫后静脉/比目鱼肌或腓肠肌肌肉静脉）：每周监测血管彩超至症状缓解，一旦血栓进展至近端深静脉，则按急性近端下肢DVT处理；③急性上肢DVT：因上肢DVT继发PTE风险较低[45]，上腔静脉置入滤器的安全性和有效性证据不足[46]，不建议上腔静脉滤器置入，可密切观察至抗凝禁忌证解除后予以抗凝治疗。

无症状非导管相关DVT的处理与有症状DVT处理原则相同。

（二）HSCT后PTE的治疗[25]–[26],[44]

高危PTE：如无溶栓禁忌证，建议静脉溶栓治疗；有溶栓禁忌证或溶栓失败者，根据各中心条件，由血液科、血管外科、介入科及呼吸科共同决策，选择导管或手术取栓，之后无抗凝禁忌者进行抗凝治疗；非高危PTE：立即开始抗凝治疗。中高危PTE抗凝治疗过程中发生病情恶化，可予以溶栓治疗。

有抗凝禁忌证的急性PTE患者，如合并存在下肢DVT，可放置临时IVC，待抗凝禁忌证解除后拆除IVC并开始抗凝治疗；大面积肺栓塞患者，可根据各中心条件及患者病情，考虑选择手术取栓。

血流动力学不稳定且不能纠正的高危PTE患者，在有条件的中心，可选择静脉动脉-体外膜肺氧合（VA-ECMO）治疗。

偶然发现的无症状PTE与有症状PTE处理原则相同。

（三）HSCT后非导管相关DVT和PTE抗凝治疗疗程

推荐HSCT后DVT或PTE的患者，抗凝治疗≥ 3个月。

目前尚无前瞻随机对照研究确定HSCT后非导管相关DVT和PTE的最佳抗凝疗程，参照肿瘤患者VTE的治疗，推荐抗凝治疗至少3～6个月。抗凝治疗满3～6个月时，评估患者血栓及出血风险，确定是否延长抗凝时间。如果恶性血液病处于活动期或正在接受化疗，或MM患者正在接受IMiD治疗，建议延长抗凝治疗时间[9],[44]。

（四）HSCT后CRT的治疗

CRT可引起不适症状及导管梗阻，增加感染以及静脉狭窄风险[47]，但引起PTE的风险较低[46],[48]。CRT的治疗目标为减轻症状、抑制血栓向中心静脉扩展或复发以及预防血栓后综合征（PTS）[42]。治疗策略包括拔除静脉导管、抗凝治疗、导管内直接溶栓及血栓清除术[31],[44],[49]。

1. 拔管指证：虽然既往研究发现，无论是否拔除静脉导管，均不影响抗凝治疗疗效[50]–[51]，但CRT增加感染风险[47]且拔除静脉导管可降低抗凝压力，对于HSCT发生CRT患者，均应积极拔除静脉导管。但如患者因生命体征不稳定、处于化疗期间、需大量补液（如腹泻）或儿童患者等原因，可酌情保留静脉导管。

2. 拔管前抗凝治疗：为防止CRT急性期拔管导致血栓脱落，如果病情允许，对于HSCT后导管相关DVT，建议在拔除输液导管前抗凝治疗3～5 d[49]。如合并不易控制的导管相关感染需要紧急拔管，或者因血小板减少（PLT<25×10^9^/L）及出血风险导致抗凝治疗受限，根据患者临床症状/体征，可缩短拔管前抗凝时间或不抗凝[49],[52]。对于导管相关SVT，由于血栓体积较小且有静脉瓣阻挡，继发PTE风险极低，可直接拔管而不必提前抗凝[49]。

3. 抗凝治疗疗程：目前缺乏HSCT后CRT治疗的前瞻性研究数据，建议参考肿瘤患者CRT的治疗[48],[51]。①如确诊导管相关DVT短期内拔除静脉导管，建议抗凝治疗3个月，如无其他VTE危险因素，抗凝治疗不必超过3个月；如留置静脉导管，建议留置期间持续抗凝治疗，且疗程不短于3个月。②导管相关SVT见SVT的治疗部分。

4. 有抗凝禁忌证导管相关DVT的治疗：因血小板减少或活动性出血等原因，无法安全实施抗凝治疗时，拔除静脉导管是防止血栓进展的有效方法，应积极拔除输液导管，并监测血栓进展。对于HSCT后血小板减少患者，导管相关DVT发生1个月内，建议根据拔管指证确定保留或拔除输液导管，输注血小板至PLT≥50×10^9^/L，并给予足量抗凝治疗（LMWH、华法林或DOAC）；如慢性DVT（病程>1个月）或血小板难以获得或血小板输注无效，PLT（25～50）×10^9^/L的患者，给予半量LMWH抗凝；PLT<25×10^9^/L或合并其他抗凝禁忌的患者，予拔除静脉输液导管、暂停抗凝并密切监测血栓进展[49],[53]。

5. HSCT后CRT的溶栓治疗适应证：不建议对HSCT后CRT常规进行溶栓治疗，HSCT后CRT进行导管直接药物溶栓治疗的适应证：急性血栓形成症状严重，出现上腔静脉综合征，肢体明显肿胀，甚至有导致骨筋膜室综合征风险和（或）抗凝治疗无效的难治性血栓形成[43],[54]。由血液科、血管外科专家共同会诊，进行导管内直接溶栓治疗。

6. HSCT后无症状CRT的处理：无症状CRT的发生率远高于症状性CRT[55]–[56]，但无症状CRT大多为附壁血栓或浅静脉血栓[56]，血栓体积小，并发肺栓塞及PTS风险较低[55]，目前无证据证明抗凝治疗可改善无症状CRT的预后[54],[56]，尤其无症状上肢CRT。因此，应充分评估患者血栓进展/复发风险与出血风险，制定无症状HSCT后CRT患者的个体化治疗方案。

（五）HSCT后SVT的治疗

HSCT后SVT（尤其是上肢SVT）绝大多数与静脉导管相关，拔管指征同上。首先对症缓解炎症刺激引起的疼痛，如保暖及抬高患肢及局部非甾体类抗炎药（NSAID）抗炎等，同时监测血栓进展。如血栓进展或血栓距离深静脉系统<3 cm，抗凝治疗45 d；下肢SVT长度>5 cm或扩展至膝关节以上，抗凝治疗45 d；下肢SVT长度<5 cm或在膝关节以下7～10 d后复查彩超，如彩超提示进展，开始抗凝治疗；下肢SVT距离股隐交界<3 cm，抗凝治疗3个月[44],[57]。

HSCT后SVT抗凝治疗达到最短疗程后进行评估，有以下VTE进展或复发的高危因素时可延长抗凝时间：抗凝治疗没有解决血栓相关的症状；存在多个血栓和（或）非CRT；初始治疗已经抗凝和拔除静脉输液导管，但血栓仍在进展或没有缓解；恶性血液病活动期；正在接受可能导致VTE风险升高的治疗[58]。

（六）抗凝治疗药物选择

抗凝治疗可选择的药物包括非口服抗凝剂（普通肝素、LMWH或磺达肝素）、维生素K拮抗剂（华法林）、DOAC（利伐沙班、阿哌沙班、艾多沙班、达比加群），具体治疗剂量及注意事项见附表4。HSCT患者的VTE治疗应首选LMWH，可序贯口服华法林或DOAC。但鉴于华法林可能影响骨髓微环境及造血重建[59]，在移植早期应尽量避免应用。如血小板计数≥50×10^9^/L，DOAC是可供选择的初始治疗或序贯口服抗凝治疗[60]，但需注意利伐沙班与细胞色素P450（CYP）3A4强效抑制剂（伊曲康唑、伏立康唑、泊沙康唑）或P糖蛋白（P-gp）抑制剂（如环孢素A）合用时会增加利伐沙班血药浓度，使用时酌情减量；艾多沙班、达比加群与环孢素A合用时剂量需减半。HSCT后SVT如有抗凝治疗适应证，可选择预防剂量抗凝药物[57]。抗凝治疗前需进行血常规、凝血分析、肌酐及肝功能检测，在抗凝治疗最初14 d，每2～3 d检测1次血常规，之后每2周检测1次或根据临床需求检测。

**附表4:** 静脉血栓栓塞症（VTE）抗凝药物治疗方案

药物名称	用法与用量
普通肝素	负荷剂量80 U/kg静脉注射，继以每小时18 U/kg静脉输注，治疗目标为APTT达到正常值的1.5～2.5倍
达肝素钠	100 U/kg每日2次或200 U/kg每日1次，皮下注射
依诺肝素	1 mg/kg每日2次或1.5 mg每日1次，皮下注射
磺达肝癸钠	体重<50 kg者5 mg，50～100 kg者7.5 mg，>100 kg者10 mg，每日1次，皮下注射
华法林	初始剂量2.5～3.0 mg每日1次口服，调整剂量使INR维持2.0～3.0；初始治疗与非口服抗凝剂联合，若连续2次INR 2.0～3.0，则停用非口服抗凝剂，继续使用华法林（一般至少需要5～7 d）
阿哌沙班	10 mg，每12 h 1次×7 d，口服；之后5 mg，每12 h 1次
利伐沙班	15 mg每日2次口服，连续21 d；之后20 mg每日1次口服（CrCl<30 ml/min者禁用）。细胞色素P450（CYP）3A4强效抑制剂（伊曲康唑、伏立康唑、泊沙康唑）或P-糖蛋白抑制剂（如环孢素A）会增加利伐沙班血药浓度，使用时酌情减量、监测出血风险
艾多沙班	先使用非口服抗凝剂5～10 d，然后序贯艾多沙班60 mg每日1次口服；CrCl 30～50 ml/min、体重<60 kg或使用P-糖蛋白抑制剂者，30 mg每日1次口服
达比加群	先使用非口服抗凝剂5～10 d，然后序贯达比加群150 mg每日2次口服；CrCl 30～50 ml/min者，75 mg每日2次口服；不建议与P-糖蛋白抑制剂（如环孢素A）合用

注：INR：国际标准化比值；CrCl：肌酐清除率；APTT：活化部分凝血活酶时间

（七）抗凝治疗禁忌证[58]

1. 绝对禁忌证：①急性重度出血（活动性出血需要输血量>2 U红细胞，HGB降幅≥20 g/L，颅内或蛛网膜下腔出血）；②留置硬膜外导管，硬膜外麻醉/腰椎穿刺（停用预防剂量LMWH 12 h内或停用治疗剂量LMWH 24 h内）

2. 相对禁忌证：①慢性、有临床意义的出血超过48 h，血小板减少［PLT<（25～50）×10^9^/L］；②潜在出血性疾病，例如排除狼疮抗凝物或抗凝治疗影响的PT或APTT延长；③已知尚未接受替代治疗的出血性疾病（如血友病）；④严重血小板功能异常；⑤近期出血风险较高的大手术；高跌倒风险（头部外伤）；⑥中枢神经系统白血病等颅内恶性肿瘤；⑦长期抗血小板治疗（对于长期接受抗血小板治疗的患者，应重新评估抗血小板治疗的必要性，尽可能停止或减少抗血小板治疗药物的剂量）。

（八）HSCT相关血小板减少的抗凝治疗

HSCT后多种原因会导致血小板减少[36]。为确定治疗策略，需要评估：①血小板减少的可能原因（GVHD、血小板重建延迟、移植相关血栓性微血管病、病毒感染、药物等，如肝素或LMWH抗凝治疗过程中出现的血小板减少，需要排除肝素诱发的血小板减少症）；②血小板减少的严重程度；③血小板减少预计持续时间；④是否可去除导致血小板减少的病因；⑤是否有凝血功能异常、活动性消化性溃疡等其他出血危险因素。目前关于血小板减少患者抗凝治疗安全性的证据有限[53],[61]–[64]。HSCT后合并血小板减少的患者，需评估抗凝治疗的风险及收益，并结合患者意愿制定个体化抗凝策略。目前缺乏DOAC用于血小板减少患者的研究数据，因此血小板减少患者首选半衰期较短的LMWH抗凝。PLT≥50×10^9^/L时，可选择DOAC抗凝治疗[60]，可供参考的抗凝治疗方案（附表5）如下：

**附表5:** 血小板减少时依诺肝素剂量调整[53], [61]–[64]

血小板计数（×10^9^/L）	剂量
>50	足量（1 mg/kg每日2次或1.5 mg/kg每日1次）
25～50	半量（0.5 mg/kg每日2次）
<25	暂停抗凝，对于VTE复发风险高和预计长期血小板减少的患者，可输注血小板以维持PLT>25×10^9^/L，保证依诺肝素的使用

注：VTE：静脉血栓栓塞症。其他低分子量肝素照依诺肝素减量比例减量

1. 血栓复发高危患者（发病1个月以内的近端DVT或PTE、复发性血栓形成）：输注血小板维持PLT≥50×10^9^/L并进行足量抗凝治疗；如血小板输注困难或输注无效，可放置可回收下腔静脉滤器，暂停抗凝[53]。

2. 血栓复发低危患者（下肢近端DVT或PTE治疗>1个月、导管相关DVT、上肢DVT、急性下肢远端DVT）：如PLT<25×10^9^/L，暂停抗凝；如PLT（25～50）×10^9^/L，予半量LMWH抗凝；如PLT>50×10^9^/L，可足量LMWH抗凝[53],[61]–[64]。导管相关DVT，予拔除静脉输液导管。急性下肢远端DVT在暂停抗凝期间，需连续监测血管彩色多普勒超声，如血栓进展至近端深静脉，按急性下肢近端DVT处理。

（九）HSCT后VTE的中医药治疗

有条件的单位，HSCT后VTE可使用水蛭为主的活血化瘀类中药复方治疗，也可使用单味菲牛蛭冻干粉预防及治疗。

六、HSCT后VTE及抗凝治疗并发症

（一）出血

HSCT患者进行抗凝治疗时出血风险升高[7],[35]–[36]，合并高龄、肝功能或肾功能不全、既往出血史、血小板减少、合并用药中有导致出血风险升高药物等出血危险因素[65]的患者，以及单倍型造血干细胞移植后消化道出血高危风险（表7）[66]的患者，在选择抗凝治疗时更应考虑出血风险。

**表7:** 单倍型造血干细胞移植后消化道出血风险预测模型[66]

项目	评分
Ⅲ/Ⅳ度急性移植物抗宿主病	1
急性肾损伤	1
移植前胃肠道疾病或出血	1
血栓性微血管病	2
弥漫性血管内凝血	2

注：0～1分为低危，2～3分为中危，4～7分为高危

出血患者首先暂停抗凝治疗，了解末次抗凝治疗时间，检测血小板计数、凝血功能、血肌酐清除率及血红蛋白水平，监测生命体征，结合出血严重程度采取相应的治疗措施：轻度出血（瘀点瘀斑、黏膜渗血），予局部止血，如仍需抗凝，出血缓解后调整抗凝药物种类和剂量，减少合并用药中可能导致出血的药物；非致命性大出血（如未影响生命体征的消化道出血），针对出血进行对症及对因治疗，可使用抗凝药物拮抗剂；致命性出血（影响生命体征的出血或脑出血），立即停用抗凝剂，使用抗凝药物拮抗剂，局部止血、输血、补液等对症支持治疗。抗凝药物拮抗剂见附表6。

**附表6:** 常用抗凝药物拮抗剂

药物名称	体内半衰期（h）	拮抗剂
普通肝素	1	鱼精蛋白1mg/100 U普通肝素，缓慢静滴（≤5 mg/min），最大剂量50 mg
		监测APTT，并需考虑到普通肝素半衰期1 h
LMWH	5～7	给药8 h内：鱼精蛋白1 mg/100 U那曲肝素或1 mg依诺肝素或100 U达肝素 给药8～12 h：鱼精蛋白0.5 mg/100 U那曲肝素或1 mg依诺肝素或100 U达肝素 给药>12 h：一般不再需要注射鱼精蛋白，如肾功能异常、出血严重等，根据个体情况可考虑给予鱼精蛋白 鱼精蛋白对LMWH的中和作用很短暂，可能需要将鱼精蛋白总量分2～4次注射，缓慢静脉滴注，最大剂量50 mg
			华法林	20～60	INR 4.5～10且无出血：暂停华法林；筛查与华法林相互影响的食物或药物，并尽量停用；排查急性肝损伤；监测INR，住院患者至少每日1次，出院患者每1～2 d 1 次；当INR降至<4时，重新开始华法林治疗，如果没有影响华法林的因素或因素不能解除，华法林减量10％～20％。4～7 d后重新检测INR，每周调整华法林剂量，直至INR稳定达标 INR>10且无出血：暂停华法林，有高危出血风险患者，予口服维生素K_1_ 1～2.5 mg，24 h后可重复给药1次，如口服维生素K_1_难以获得或口服困难，维生素K_1_也可缓慢静脉注射（<1 mg/min） 需要进行急诊手术（24～48 h内）：①24 h内手术：暂停华法林，维生素K_1_ 1～1.5 mg缓慢静脉注射，术前复查INR确定是否需给予FFP。②48 h内手术：暂停华法林，给予口服维生素K_1_ 2.5 mg，24、48 h后分别复查INR，确定是否再给予维生素K_1_或FFP。③严重出血（不必考虑INR）或威胁生命的出血：暂停华法林、给予维生素K_1_ 10 mg缓慢静脉注射；给予PCC 25～50 U/kg+ FFP 2～3 U；密切监测INR，必要时重复给予PCC或FFP；如PCC难以获得或止血疗效不佳，可予rhFⅦa 25 µg/kg；密切监测INR
						磺达肝癸钠	17～21	停止用药，无拮抗剂；rhFⅦa 90 µg/kg静脉注射，根据出血症状给药
						利伐沙班	9～12（老年人延长）	停止服用药物，服药2 h内使用活性炭减少其吸收，6 h后可重复；特异性拮抗剂ANDEXXA（重组凝血因子Ⅹa，中国尚未上市）
阿哌沙班	12	同上
艾多沙班	10～14	同上
达比加群	13.4	停止服用药物，使用特异性拮抗剂依达赛珠单抗（Idarucizumab）；如拮抗剂难以获得，可选择透析治疗，但经验有限

注：INR：国际标准化比值；LMWH：低分子量肝素；FFP：新鲜冰冻血浆；PCC：凝血酶原复合物；rhFⅦa：重组人凝血因子Ⅶa；血小板减少、血小板功能异常、凝血酶活性下降、合并出血等情况下，鱼精蛋白可加量至1.3 mg/100 U普通肝素或LMWH。直接口服抗凝剂（DOAC）引起的严重出血，均可考虑使用PCC或rhFⅦa治疗

（二）感染

CRT容易继发导管相关血流感染[47]。HSCT后免疫功能下降，感染发生率升高。HSCT后CRT时，由于血液瘀滞或导管不通畅，导管微生物定植，导管相关的感染容易发生。可疑导管相关感染时即应拔管，积极抗感染治疗。

（三）PTS

及时、充分的抗凝治疗是预防PTS最有效的措施。不建议对所有VTE患者使用分级弹力加压袜等常规进行PTS预防，前瞻性双盲临床研究未证实分级弹力加压袜可降低PTS风险[67]。

七、HSCT后VTE的预后

VTE不仅导致HSCT患者生活质量下降，对长期生存也有不良影响[1]–[2]。重视HSCT患者的VTE防治对于患者保障医疗安全和改善预后具有重大意义。
